# Novel biomarkers of resistance of pancreatic cancer cells to oncolytic vesicular stomatitis virus

**DOI:** 10.18632/oncotarget.11202

**Published:** 2016-08-11

**Authors:** Eric Hastie, Marcela Cataldi, Megan J. Moerdyk, Sébastien A. Felt, Nury Steuerwald, Valery Z. Grdzelishvili

**Affiliations:** ^1^ Department of Biological Sciences, University of North Carolina at Charlotte, Charlotte, NC, USA; ^2^ Cannon Research Center, Carolinas Healthcare System, Charlotte, NC, USA

**Keywords:** pancreatic cancer, oncolytic virus, vesicular stomatitis virus, interferon-stimulated gene, biomarker of resistance

## Abstract

Vesicular stomatitis virus (VSV) based recombinant viruses (such as VSV-ΔM51) are effective oncolytic viruses (OVs) against a majority of pancreatic ductal adenocarcinoma (PDAC) cell lines. However, some PDAC cell lines are highly resistant to VSV-ΔM51. We recently showed that treatment of VSV-resistant PDAC cells with ruxolitinib (JAK1/2 inhibitor) or TPCA-1 (IKK-β inhibitor) breaks their resistance to VSV-ΔM51. Here we compared the global effect of ruxolitinib or TPCA-1 treatment on cellular gene expression in PDAC cell lines highly resistant to VSV-ΔM51. Our study identified a distinct subset of 22 interferon-stimulated genes (ISGs) downregulated by both ruxolitinib and TPCA-1. Further RNA and protein analyses demonstrated that 4 of these genes (MX1, EPSTI1, XAF1, and GBP1) are constitutively co-expressed in VSV-resistant, but not in VSV-permissive PDACs, thus serving as potential biomarkers to predict OV therapy success. Moreover, shRNA-mediated knockdown of one of such ISG, MX1, showed a positive effect on VSV-ΔM51 replication in resistant PDAC cells, suggesting that at least some of the identified ISGs contribute to resistance of PDACs to VSV-ΔM51. As certain oncogene and tumor suppressor gene variants are often associated with increased tropism of OVs to cancer cells, we also analyzed genomic DNA in a set of PDAC cell lines for frequently occurring cancer associated mutations. While no clear correlation was found between such mutations and resistance of PDACs to VSV-ΔM51, the analysis generated valuable genotypic data for future studies.

## INTRODUCTION

Oncolytic virus (OV) therapy using replication-competent viruses has shown preclinical success against many malignancies, and some OVs are now approved for use in the United States and Latvia for melanoma [1, 2] and in China for head and neck squamous cell carcinoma [3]. Vesicular stomatitis virus (VSV) is efficacious against various cancer types in preclinical studies and is currently in a phase I clinical trial against hepatocellular carcinoma (trial NCT01628640) [4]. Our work focuses on VSV-ΔM51, a recombinant VSV with methionine deleted at position 51 in the VSV matrix (M) protein [5]. The ΔM51 mutation ablates wild type (WT) M protein's ability to inhibit cellular antiviral gene expression [6–8], while still allowing VSV to replicate in and kill cancer cells, as many cancers have defective type I interferon (IFN) antiviral responses. Importantly, the ΔM51 mutation also strongly inhibits neurotoxicity associated with WT VSV, and VSVs with different ΔM51 mutations have been explored extensively [4, 9, 10].

This work focused on pancreatic cancer, the fourth leading cause of cancer-related deaths worldwide [11]. Pancreatic ductal adenocarcinoma (PDAC) is the most common pancreatic neoplasm. Lack of early detection, aggressive local metastases, and limited treatment options means PDAC diagnosis closely mirrors mortality. Surgical removal of tumors is possible in less than 20 percent of patients and current chemo or radiation-based therapies fail to significantly extend life expectancy [12]. Various OVs have been tested against PDAC *in vitro* and *in vivo* with limited efficacy [13]. An understanding of the cellular factors that prevent or allow success is lacking.

The use of VSV-ΔM51 against human PDAC cell lines *in vitro* and *in vivo* demonstrated its therapeutic promise [14]. However, while VSV-ΔM51 kills a majority of human PDAC cell lines *in vitro*, resistance of some cell lines to this virus needs to be addressed [14, 15]. Our previous studies showed that not only resistant but many permissive PDAC cell lines are able to mount type I IFN responses, producing type I IFNs and IFN-stimulated genes (ISGs) in response to VSV-ΔM51 infection [14, 15]. However, only resistant cell lines showed high-level constitutive expression of the ISGs MX Dynamin-Like GTPase 1 (MX1) and 2′-5′-Oligoadenylate Synthetase 2 (OAS2) [15]. We also demonstrated that resistance of PDAC cell lines to VSV-ΔM51 can be overcome by combining virus with IFN signaling inhibitors such as Janus kinase (JAK) inhibitor I and ruxolitinib [15, 16]. In addition, we showed a similar effect for TPCA-1 [16], which had previously been described as a direct inhibitor of IKK-β [17–19]. Our study demonstrated [16] pleiotropy for TPCA-1, which inhibited not only IKK-β [17–19], but also JAK1 kinase activity [16].

The goal of the current study was to further elucidate the role of ruxolitinib and TPCA-1 in breaking resistance of PDACs to VSV-ΔM51, and to identify gene expression signatures of PDAC resistance to VSV-ΔM51, which could serve as potential biomarkers to predict OV therapy success. The gene expression profiling was the first ever analysis of the global effects of ruxolitinib or TPCA-1 on PDAC transcriptomes, and allowed for further comparison of the molecular mechanisms of action of these drugs. Our study identified a set of 8 ISGs as putative biomarkers of PDAC resistance to VSV-ΔM51, and our data suggest that at least some of the identified ISGs contribute to resistance of PDACs to VSV-ΔM51. Importantly, 4 of these 8 putative biomarkers have never been studied in regard to VSV infection, thus representing potential novel cellular factors restricting VSV replication. Additionally, as certain variants of oncogenes and tumor suppressor genes are often associated with increased tropism of OVs to cancer cells (e.g., by affecting type I IFN signaling regulation), we also conducted a genomic analysis of PDAC cell lines for frequently occurring cancer mutations.

## RESULTS

### Effect of ruxolitinib and TPCA-1 on transcriptomes of PDAC cell lines

**Figure 1:**
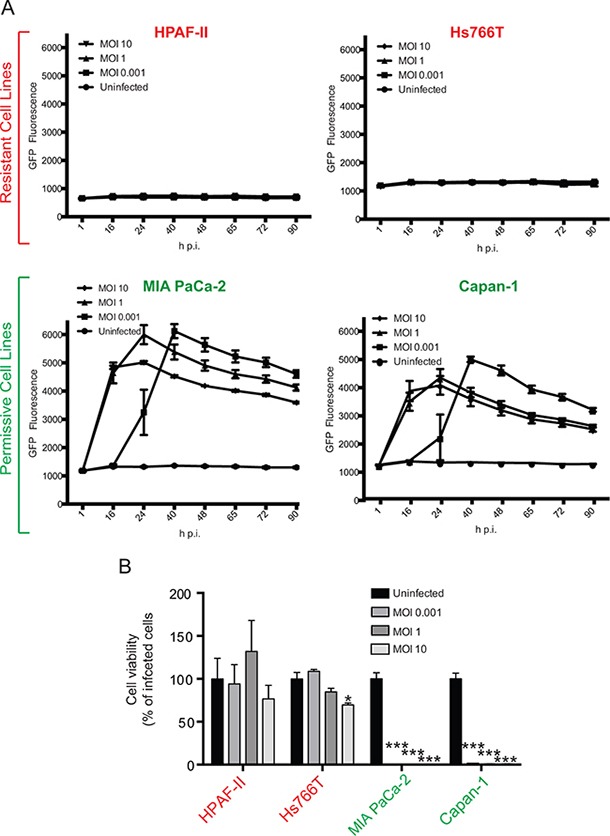
Phenotypes of VSV-permissive and VSV-resistant PDAC cell lines VSV-ΔM51 replication **A.** and VSV-ΔM51-mediated oncolysis **B.** in 4 different human PDAC cell lines. Cells were infected with VSV-ΔM51 at 3 different MOIs (0.001, 1, or 10) or mock-treated, and (A) virus replication driven GFP fluorescence was monitored through 90 h p.i. and (B) cell viability was analyzed by MTT assay at 90 h p.i. and is plotted as percentage of the uninfected control. The assays were done in triplicate and data represent the mean±SEM. Statistical analysis was performed using GraphPad Prism Software, using one-way ANOVA with Bonferroni post-test for comparison to the control. (*) P<0.01; (***) P<0.0001 (*) indicate statistical significance between infected and uninfected cells within the same cell line.

Our previous studies showed that while most of the tested human PDAC cell lines are permissive to VSV-ΔM51, some are highly resistant to this virus [14, 15, 20]. The current study is focused on two permissive PDACs, MIA PaCa-2 and Capan-1, and two resistant PDACs, HPAF-II and Hs766T. As cancer cell can be genotypically and phenotypically unstable, we reexamined permissiveness of these 4 PDAC cell lines to VSV-ΔM51. MIA PaCa-2, Capan-1, HPAF-II, and Hs766T were infected with VSV-ΔM51 at a range of MOIs (calculated based on VSV-ΔM51 titer on BHK-21, a reference cell line highly permissive to VSV), and monitored for GFP expression to measure virus replication kinetics (Figure 1A), and for virus-mediated oncolysis using MTT cell viability analysis (Figure 1B). Consistent with previous observations, Hs766T and HPAF-II showed strong resistance to VSV as extremely limited GFP was detected at all time points (Figure 1A) and practically no cell death occurred even at the highest tested MOI (Figure 1B). In contrast, MIA PaCa-2 and Capan-1 cell lines were permissive to VSV-ΔM51 as GFP was readily detectable at most time points (Figure 1A), and all cells were dead by the endpoint at all tested MOIs (Figure 1B).

By examining expression of select antiviral ISGs by RT-PCR, we previously showed that only resistant cell lines showed high-level constitutive expression of MX1 and OAS2 [15]. Furthermore, inhibition of type I IFN signaling by ruxolitinib or TPCA-1 dramatically improved VSV-ΔM51 replication and PDAC killing while decreasing expression of these ISGs in both infected and uninfected PDACs [16]. Despite having very similar effects on VSV-ΔM51 in PDAC cells, these two drugs differ in target specificity, with ruxolitinib targeting both JAK1 and JAK2 [16, 21], and TPCA-1 targeting IKK-β [17–19], JAK1 [16], and possibly other JAKs. As these drugs are capable of inhibiting pathways other than just type I IFN signaling, determining the global impact of these drugs on PDAC cells is a key step in identifying the mechanism(s) of resistance to VSV-ΔM51 and the role of these drugs in breaking that resistance. To compare the global effects of ruxolitinib and TPCA-1 on cellular gene expression, microarray analysis was performed on both untreated and ruxolitinib or TPCA-1 treated HPAF-II and Hs766T, the two cell lines with the strongest resistance to VSV-ΔM51. Cells were treated with 2.5 μM ruxolitinib or 8 μM TCPA-1 as these doses show the greatest effect on VSV replication without significant drug-mediated toxicity [16]. Total RNA was collected at 6 h post-treatment as in the absence of treatment VSV replication is already severely impaired by 6 h p.i., suggesting that the relevant targets are downregulated rapidly [14, 16]. Furthermore, by choosing a relatively early time point we hoped to primarily capture changes in gene expression directly affected by the drug treatment. Total RNA was collected and reverse transcribed for use with Affymetrix Human Genome U133+ Plus PM array strips, which report on expression levels of more than 47,000 transcripts and variants selected from GenBank, dbEST, and RefSeq. The threshold for our initial analysis was set so that only transcripts with a 2-fold expression level change or greater at least in one sample were included. For transcripts identified at the 2-fold or greater level at least in one sample, expression at the 1.5-fold level was also determined for the remaining samples.

As HPAF-II and Hs766T have similar phenotypes in regard to both VSV replication and ISG expression, we first examined whether these two cell lines had a similar transcription profile. Comparing untreated HPAF-II to untreated Hs766T cells identified 6932 probes representing 4196 genes with at least a 2-fold difference in mRNA expression, with some genes showing more than a 400-fold difference in expression (Supplementary Table S2). These genes include many with known impacts on PDAC biology and clinical outcome [22–24] including CEACAM5 (153-fold difference), ERBB3 (62-fold), CLDN4 (59-fold), LCN2 (27-fold), FN1 (20-fold), TGFBI (19-fold), PLAUR (17.5-fold), LAMC2 (8.6-fold), CAPG (8-fold difference), AGR2 (7-fold difference), FXYD3 (5.3-fold), LAMC1 (5.1-fold), MUC1 (4.6-fold), MUC4 (4.4-fold), ITGA1 (3.6-fold), COTL1 (2.5 fold), CD9 (2.3-fold), ITGA3 (2.3-fold), TP53 (2.2-fold), MMP14 (2.1-fold). These data demonstrate that PDAC cell lines with dramatic differences in their transcriptome could show very similar phenotypes in regard to VSV resistance and the drugs breaking that resistance.

Ruxolitinib treatment of either HPAF-II or Hs766T affected the expression of a surprisingly small number of RNAs with 26 probes representing 20 genes and 32 probes representing 28 genes respectively changing by 2-fold or more upon treatment (Table 1; full listing of probes in Supplementary Table S3). Nearly all identified genes were downregulated and the majority are documented ISGs as based on the Interferome Database v2.01 (see Methods). This list includes MX1, which was identified in our previous studies as constitutively expressed in VSV resistant cell lines [15], but also a number of ISGs where expression was not previously examined. There is a high degree of overlap in the genes differentially expressed in both HPAF-II and Hs766T with 9 of 38 genes with at least a 2-fold difference in expression being shared. For the remaining genes with a minimum of a 2-fold difference in only one cell line, the change in expression in the other cell line was determined to be at least 1.5-fold for most ISGs, but not necessarily for the other gene classifications (Table 1; complete listing of genes changed at the 1.5-fold threshold is given in Supplementary Table S4). This suggests that downregulation of a relatively small and consistent set of ISGs is associated with ruxolitinib treatment of two different VSV resistant PDACs. These data are consistent with ruxolitinib as a specific JAK1/JAK2 inhibitor.

**Table 1:** All genes with at least a 2-fold change in expression upon Ruxolitinib treatment in at least one cell type

	Entrez Gene	Gene Symbol	RefSeq Transcript ID^a^	Fold-Change HPAF-II + Ruxolitinib^c^,^d^	Fold Change Hs766T + Ruxolitinib^c^,^d^	Fold Change HPAF-II + TPCA^c^,^d^	Fold Change Hs766T + TPCA^c^,^d^
**Type I Interferon Regulated**	80830	APOL6	NM_030641	*−1.702*	−2.121/*−1.561*	-	-
	129607	CMPK2	NM_207315	−4.166	−2.216	−3.307	−2.141
	23586	DDX58	NM_014314	*−1.837*	−2.016/*−1.743*	*−1.883*	−2.304/*−1.957/−1.705*
	55601	DDX60	NM_017631	−2.007	-	−2.255	-
	151636	DTX3L	NM_138287	-	−2.571	*−1.868*	−3.484
	94240	EPSTI1	NM_001002264^b^	−2.079	*−1.970/−1.960*	*−1.966*	−2.042/−*1.683*
	2633	GBP1	NM_002053	*−1.545*	−2.100/*−1.982/−1.687*	*−1.589/−1.560/−1.509*	−2.349/−2.214/−2.140
	10561	IFI44	NM_006417	−3.119/*−1.937*	−3.470/*−1.831*	−3.600/−2.268	−6.353/*−1.914*
	10964	IFI44L	NM_006820	−3.530	−2.436	−2.994	*−1.593*
	64135	IFIH1	NM_022168	−2.123	*−1.885/−1.564*	−2.160	−2.199/*−1.661*
	3434	IFIT1	NM_001548	−6.515	−2.756	−6.448	−3.104
	3433	IFIT2	NM_001547	−3.314/−2.626	−2.085	−2.676/*−1.987*	*−1.731*
	3437	IFIT3	NM_001031683^b^	−4.442/−2.577	−2.531/−2.142	−3.928/−2.771	−2.416/−2.301
	3659	IRF1	NM_002198	-	−2.086	*−1.696*	−3.357/*−1.937*
	3665	IRF7	NM_001572^b^	−2.094	-	−2.329	-
	10379	IRF9	NM_006084	*−1.678*	−3.855	-	−2.314
	27074	LAMP3	NM_014398	−2.055	-	*−1.704*	-
	4599	MX1	NM_001144925^b^	−2.929	−2.354	−2.941	−2.863
	4600	MX2	NM_002463	−4.578	*−1.881*	−3.863	*−1.853*
	4939	OAS2	NM_001032731^b^	−3.144/−2.867/−2.055	*−1.933/−1.879*	−3.225/−2.801/−2.205	−2.297/*−1.954*
	83666	PARP9	NM_001146102^b^	−2.013/*−1.602*	−3.402/*−1.877*	−2.582/−2.161	−4.154/*−1.982*
	91543	RSAD2	NM_080657	−4.598/−4.156	*−1.715/−1.584*	−3.885/−3.347	-
	64108	RTP4	NM_022147	*−1.971*	−2.133	−2.997	−2.233
	219285	SAMD9L	NM_152703	*−1.811*	−2.684/−2.019/ *−1.748*	−2.587/−2.231/ *−1.902*	−3.095/−3.044/ −2.329
	3431	SP110	NM_001185015^b^	*−1.990/−1.881/ −1.750/−1.719*	−2.222/−2.211/ *−1.911/−1.872/ −1.656*	−2.324/−2.189/ −2.019/*−1.941/ −1.548*	−3.323/−2.467/ −2.009/*−1.948/ −1.905*
	10346	TRIM22	NM_006074	−2.441	-	−2.287	*−1.592*
	11274	USP18	NM_017414	−2.787	−3.051	−3.148	−3.826
	54739	XAF1	NM_017523^b^	−2.668/−2.450	−3.312/−2.608	−2.579/−2.149	−2.544/*−1.953*
**Non-mRNA**	---	---	235157_PM_at	*−1.874*	−2.118	−2.560	−2.602
	---	---	243271_PM_at	-	−2.324	-	−2.811
	---	---	232375_PM_at	−2.345	-	−2.289	-
**Other**	358	AQP1	NM_001185060^b^	-	2.164	-	*−1.936*
	11067	C10orf10	NM_007021	-	−2.190	-	-
	135398	C6orf141	NM_001145652	-	2.336	-	5.384/2.050
	90865	IL33	NM_033439	-	2.077	-	-
	55180	LINS	NM_001040616	-	2.073	*1.611*	2.358
	6646	SOAT1	NM_003101	-	2.057	-	*−1.521*
	80351	TNKS2	NM_025235	-	3.422	-	2.370

Changes in expression of those same genes upon TPCA treatment is also indicated.

a Probeset ID given for non-mRNAs

b Matches more than one transcript variant; see Supplementary Table S3 for full list of transcripts

c Multiple values indicate multiple probes; see Supplementary Table S3 for list of probes

d For treatments where the change is expression was less than 2-fold: changes greater than 1.5-fold are indicated in italics (see Supplementary Table S4 for full listing); changes less than 1.5-fold are indicated by “-”

As expected for a drug that targets not only type I IFN signaling but also NF-kB signaling, TPCA-1 affected a much larger number of genes with 260 probes representing 226 genes for HPAF-II and 422 probes representing 348 genes for Hs766T at the 2-fold level (Supplementary Table S3). A number of these genes are known ISGs and many of these are the same genes differentially expressed in response to ruxolitinib treatment, with eight being shared in common between all four cell-treatment combinations (CMPK2, IFI44, IFIT1, IFIT3, PARP9, USP18, XAF1) and an additional eight between three of the treatments at the 2-fold level (Table 1). Expression of these genes, individually or together, may serve as biomarkers for resistance of PDAC cells to VSV-ΔM51. Several additional ISGs were also identified as being changed at least 2-fold in response to TPCA-1 treatment, but that did not reach that threshold with ruxolitinib (Table 2), although several of these genes were affected at least 1.5-fold in response to ruxolitinib treatment (Table 2 and Supplementary Table S4).

**Table 2:** ISGs with at least a 2-fold change in expression upon TPCA but not Ruxolitinib treatment

Entrez Gene	Gene Symbol	RefSeq Transcript ID^a^	Fold Change HPAF-II + TPCA^b^,^c^	Fold Change Hs766T + TPCA^b^,^c^	Fold-Change HPAF-II + Ruxolitinib^b^,^c^	Fold Change Hs766T + Ruxolitinib^b^,^c^
55337	C19orf66	NM_018381	−2.766/*−1.739*	-	*−1.676*	-
6347	CCL2	NM_002982	-	−3.424	-	*−1.736*
24138	IFIT5	NM_012420	*−1.841/−1.665*	−2.033/*−1.963*	*−1.820/−1.784*	*−1.927/−1.883*
4938	OAS1	NM_001032409^a^	*−1.909/−1.771*	−2.677/*−1.758*	*−1.717/−1.676*	-
8638	OASL	NM_003733^a^	−2.490/−2.250	-	*−1.882/−1.720*	-
57674	RNF213	NM_020914^a^	−2.240/*−1.604*	-	*−1.886/−1.505*	-
54809	SAMD9	NM_001193307^a^	−2.215/−2.142	−2.594/−2.308	-	*−1.64*
11277	TREX1	NM_016381^a^	−2.111	*−1.885*	-	-
9830	TRIM14	NM_014788^a^	*−1.754/−1.540/ −1.503*	−2.316/−2.181	-	*−1.886/−1.772*
6737	TRIM21	NM_003141	-	−2.362	-	*−1.658*

a Matches more than one transcript variant; see Supplementary Table S3 for full list of transcripts

b Multiple values indicate multiple probes; see Supplementary Table S3 for list of probes

c For treatments where the change is expression was less than 2-fold: changes greater than 1.5-fold are indicated in italics (see Supplementary Table S4 for full listing); changes less than 1.5-fold are indicated by “-”

In addition to impacting ISGs, treatment with TPCA-1 also affected expression of a number of other genes, including downregulation of several genes associated with the NF-kB pathway including IL8 (Supplementary Tables S3 and S4). This supports our recent report that TPCA-1 acts as an NF-kB pathway inhibitor as well as a JAK1 inhibitor [16]. Other differentially expressed genes affected by TPCA-1 could not be obviously identified as associated with either the type I IFN or NF-kB pathways (Supplementary Tables S3 and S4).

### ISGs constitutively expressed in resistant but not permissive PDAC cell lines

**Figure 2:**
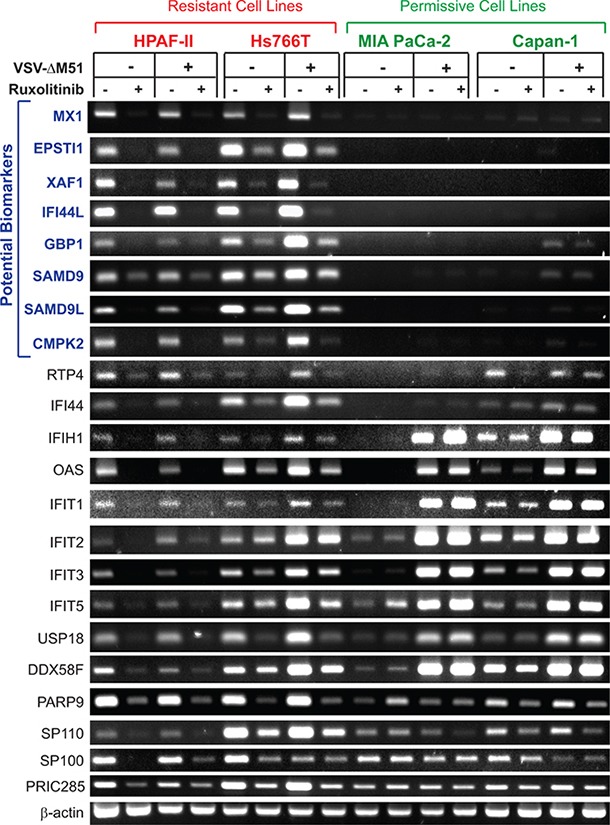
RT-PCR analysis of gene expression of putative biomarkers of resistance in PDAC cell lines Resistant cells (HPAF-II and Hs766T) and permissive cells (MIA PaCa-2 and Capan-1) were mock treated or treated with ruxolitinib (2.5 μM) for 24 h prior to mock treatment or infection with VSV-ΔM51 at MOI 10 (based on virus titer on BHK-21 cells). Virus was aspirated after 1 hour absorption and replaced with growth media containing 5% FBS. Total RNA was extracted 12 h p.i. and reverse transcribed. Gene specific primers (Supplementary Table S1) were used to amplify cDNA. PCR products were run on a 2% agarose gel. All reactions were run in duplicate/triplicate, and one representative sample is shown for each condition.

Our microarray data show that a number of ISGs are downregulated by both ruxolitinib and TPCA-1, suggesting they are constitutively expressed in uninfected HPAF-II and Hs766T. To confirm these findings and determine if they are specific to VSV-resistant HPAF-II and Hs766T cells but not to VSV-permissive MIA PaCa-2 and Capan-1 cells, and to determine whether these genes are inducible by VSV infection, these 4 cell lines were pretreated (or mock-treated) with ruxolitinib for 24 h, then infected (or mock-infected) with VSV-ΔM51 (at MOI 10) for 12 h in the presence or absence of ruxolitinib. Total RNA was isolated, reverse transcribed, and analyzed by semi-quantitative PCR. For this analysis, we selected the genes which were downregulated in both HPAF-II and Hs766T at least 1.5-fold with both ruxolitinib and TPCA-1 and at least 2-fold with at least one treatment. All 22 genes meeting these criteria were ISGs (based on the Interferome Database v2.01) and are included in Table 1. In agreement with our microarray data, all selected ISGs were constitutively expressed in VSV-resistant HPAF-II and Hs766T cells, with ruxolitinib significantly inhibiting their expression (Figure 2). Importantly, expression was generally not increased in response to VSV-ΔM51 expression (Figure 2). This profile is not seen in the 2 permissive cell lines, MIA PaCa-2 and Capan-1. Out of 22 tested genes, expression of 14 genes was detectable in at least one VSV-permissive cell line even in the absence of VSV-ΔM51. Expression of eight of these genes (IFIH1, OAS2, IFIT1, IFIT2, IFIT3, IFIT5, USP18 and DDX58F) was significantly elevated in response to VSV-ΔM51 infection in permissive cells, but not in resistant PDACs. Expression of the remaining four genes (PARP9, SP110, SP100 and PRIC285) was unchanged by infection. Importantly, no clear effect of ruxolitinib could be seen in VSV-permissive MIA PaCa-2 or Capan-1 cells. Despite these key differences, these genes are not optimal biomarkers of resistance due to their detectable expression in at least some permissive PDACs.

**Figure 3:**
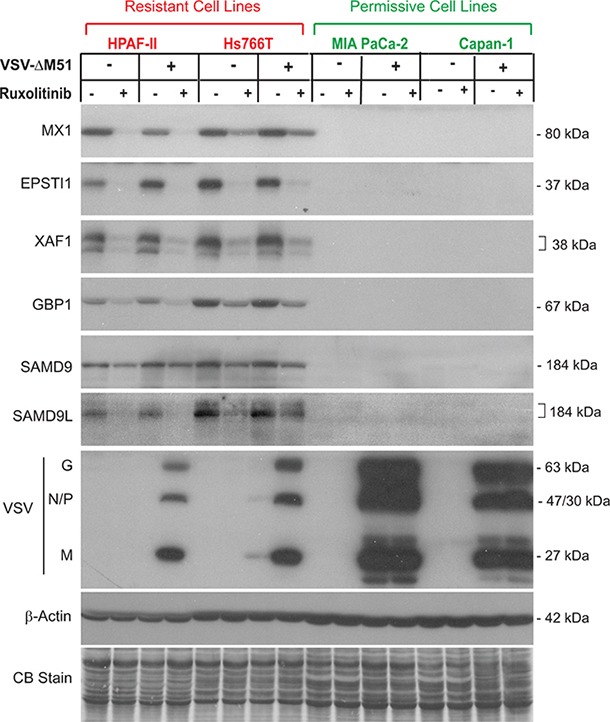
Western blot analysis of putative biomarkers of resistance in PDAC cell lines Protein expression following ruxolitinib treatment and VSV-ΔM51 infection. Cells were mock treated or treated with ruxolitinib (2.5 μM) for 24 h prior to mock treatment or infection with VSV-ΔM51 at an MOI of 10 (based on virus titer on BHK-21 cells). At 16 h p.i., cell lysates were prepared and analyzed by Western blot for the indicated protein. Protein sizes (kDa) are indicated on the right. Actin protein levels and Coomassie Blue staining of total protein demonstrate equal loading of protein.

On the other hand, 8 genes were constitutively expressed in resistant cells but showed no detectable expression in permissive MIA PaCa-2 or Capan-1 cells. These 8 genes, MX1, EPSTI1, XAF1, IFI44L, GBP1, SAMD9, SAMD9L, and CMPK2 (Figure 2), do represent candidate biomarkers of resistance of PDAC cells to VSV-ΔM51. To confirm this result at the protein level, a similar experiment was conducted but with infection for 16 h rather than 12 h to detect changes in protein accumulation. Total protein from VSV-resistant and VSV-permissive PDAC cell lines was analyzed for expression of these 8 potential biomarkers (Figure 3). Western blot analysis confirmed the transcriptome and RT-PCR analyses for six potential biomarkers of resistance: MX1, EPSTI1, XAF1, GBP1, SAMD9 and SAMD9L (Figure 3). However, compared to mRNA analyses by microarray and RT-PCR (Figure 2), we observed smaller effect of ruxolitib on SAMD9 protein by Western blot. It is very likely that the observed differences are due to the stability of the preexisting pool of SAMD9 protein that may cause significant amounts of this protein to still be present 16 h after treatment. Unfortunately, we were not able to confirm our RT-PCR results for IFI44L and CMPK2 by Western blot. Two commercial antibodies were tested for each protein (see Materials and Methods for details). For IFI44L there was no detectable signal with either antibody. For CMPK2 protein, two antibodies showed different patterns of bands of different sizes and intensities, and both patterns were inconsistent with our RNA data (data not shown).

**Figure 4:**
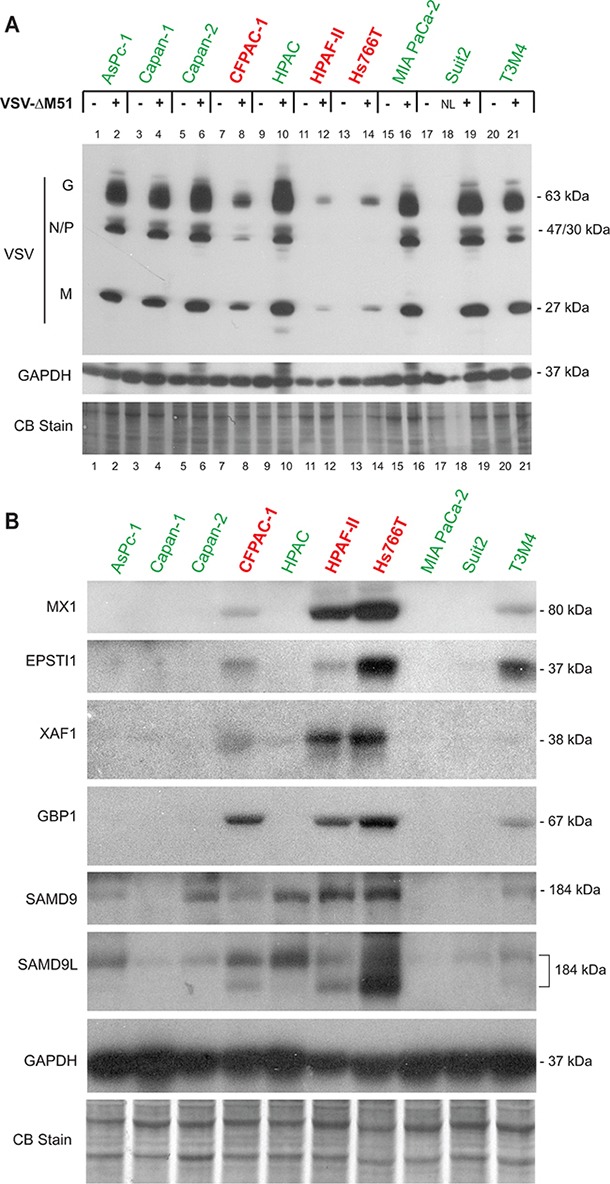
Western blot analysis of putative biomarkers of resistance in 10 PDAC cell lines **A.** Protein expression following mock-treatment or VSV-ΔM51 infection. Cells were mock treated or infected with VSV-ΔM51 at an MOI of 5 (based on virus titer on Suit-2 cells, which have an average permissiveness to VSV-ΔM51). At 8 h p.i., cell lysates were prepared and analyzed by Western blot for the indicated protein. “NL” – not loaded, the well #18 was skipped. **B.** Cells were seeded and protein was isolated 24 h later. Protein sizes (kDa) are indicated on the right. Actin protein levels and Coomassie Blue staining of total protein demonstrate equal loading of protein.

To further investigate whether the selected potential biomarkers are expressed only in resistant PDAC cell lines, we compared constitutive levels of the 6 putative biomarkers for which good antibodies were available in 3 resistant cell lines (HPAF-II, Hs766T, and CFPAC-1) and 7 permissive cell lines (Figure 4). Note that in this experiment HPAC, which in previous publications behaved as a moderately resistant cell line [14, 15], now behave as a permissive cell line while all other cell lines behave in a manner consistent with previous observations (Figure 4A). Four of the six potential biomarkers (MX1, EPSTI1, XAF1, and GBP1) were expressed only in resistant PDAC cell lines, with the exception of the T3M4 cell line, which is relatively permissive to VSV, but still shows detectable levels of MX1, EPSTI1, and GBP1 (Figure 4B). This result is consistent with our previous observation that T3M4 was the only VSV-permissive PDAC cell line that constitutively expressed MX1 even in the absence of viral replication [15]. While T3M4 can be completely killed in vitro by low MOI VSV infection (our criterion for being “permissive”), T3M4 is less susceptible to VSV infection and killing than other permissive cell lines [14]. Therefore, it is not surprising that it shares some features in common with the highly resistant cell lines. In agreement with Figure 3, the other 2 putative biomarkers, SAMD9 and SAMD9L, were expressed in highly resistant HPAF-II and Hs766T cells, but not in the highly permissive MIA PaCa-2 and Capan-1 cells. However, we also observed expression of SAMD9 and one of the bands for SAMD9L (the upper band in Figure 4B that could be associated with a posttranslational modification of the same protein or an alternative mRNA vartiant) in some VSV-permissive cell lines, albeit frequently at lower levels, indicating that these genes cannot be used individually as biomarkers of PDAC resistance to VSV. Together, the analysis of the 10 PDAC cell lines demonstrates that the 4 genes (MX1, EPSTI1, XAF1, and GBP1) are constitutively co-expressed in VSV-resistant, but not in VSV-permissive PDACs, thus serving as potential biomarkers to predict OV therapy success.

### Role of MX1 in resistance to VSV

**Figure 5:**
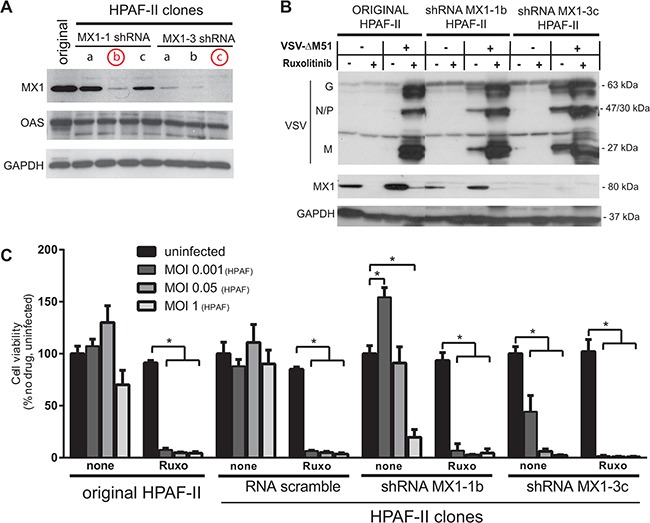
Effect of MX1 knockdown on VSV-ΔM51 replication and oncolysis **A.** HPAF-II based cell line clones were generated using a lentivector system with the vector genomes carrying MX1-shRNA1 or MX1-shRNA3 sequences. Multiple cell clones for each shRNA construct were puromycin selected and cell lysates were prepared for Western blot analysis of MX1 expression. **B.** Protein expression following ruxolitinib treatment and/or VSV-ΔM51 infection. Cells were mock treated or treated with VSV-ΔM51 at an MOI of 10 (based on virus titer on BHK-21 cells) and/or ruxolitinib (2.5 μM). At 48 h p.i., cell lysates were prepared and analyzed by Western blot for the indicated protein. Protein sizes (kDa) are indicated on the right. **C.** HPAF-II and HPAF-II based clones MX1-1b, MX1-3c and SCRA (scramble shRNA) were mock treated or treated with ruxolitinib (2.5 μM) for 24 h prior to infection with VSV-ΔM51 at an MOI of 0.001, 0.05, or 1 (based on virus titer on HPAF-II cells; 1 MOI_HPAF_ equates 1500 MOI_BHK-21_). MTT cell viability analysis was conducted 96 h p.i. The MTT assay was done in triplicate and data represent the mean±SEM. Statistical analysis was performed using GraphPad Prism Software, using multiple t-tests for comparison to uninfected control. (*) indicates statistical significance (p<0.05) between infected and uninfected cells within the same cell line.

Our data identified 8 cellular genes constitutively expressed in VSV-resistant, but not in VSV-permissive PDACs, thus serving as potential biomarkers of PDAC resistance to VSV-ΔM51 and possibly other OVs. While we have shown previously that MX1 is constitutively expressed in resistant cell lines [15], the remaining 7 genes are a novel finding regarding PDAC resistance to OV therapy. While a biomarker of resistance need not be a causative factor of resistance, it is likely that at least some of these ISGs contribute to resistance of PDACs to VSV-ΔM51, because ruxolitinib and TPCA-1 treatment, which downregulated these genes, strongly enhanced VSV-ΔM51 replication in resistant PDAC cell lines [16]. The role for each of these putative biomarkers in antiviral activity against VSV is beyond the scope of this project, especially as some of these 8 genes have been already demonstrated to have antiviral activities in other cell types. Here we focused on the potential contribution of MX1 to the resistance of HPAF-II to VSV-ΔM51. MX1 was the first potential biomarker of PDAC cell resistance identified by our group [15] and it is a known inhibitor of VSV in other systems [25, 26]. However, our earlier works only showed a correlation between MX1 expression and resistance to VSV, without examining causation, and the antiviral role of MX1 against VSV has never been examined in PDAC cells. Using a lentivector system carrying shRNA expression cassettes, we generated two HPAF-II cell lines with stable shRNA-mediated knockdown (KD) of MX1 expression (Figure 5A), MX1-1B with partial downregulation of MX1 and MX1-3C with complete (undetectable MX1 levels) downregulation of MX1.

To examine effect of MX1 downregulation on VSV-ΔM51 replication, we analyzed viral replication in MX1-1B and MX1-3C KD cell lines, and compared it to the original HPAF-II cell line. Cells were infected with VSV-ΔM51 at MOI 10 (based on VSV-ΔM51 titer on BHK-21 cells) or mock-infected, in the presence or absence of ruxolitinib. As shown in Figure 5B, in the original HPAF-II VSV replication could be detected only in the presence of ruxolitinib, whereas VSV bands could be seen in MX1-1B and MX1-3C clones even in the absence of ruxolitinib. VSV replication levels were higher in the MX1-3C clone than in the MX1-1B, likely because MX1 was completely downregulated in MX1-3C and only partially downregulated in MX1-1B. Although MX1 downregulation strongly stimulated VSV replication, this effect was markedly lower than the effect of ruxolitinib in both MX1-1B and MX1-3C clones, suggesting that that MX1 expression is only partially responsible for resistance of HPAF-II cells to VSV-ΔM51.

In a separate experiment, cells were mock treated or treated with ruxolitinib, then infected with VSV-ΔM51 at 3 different HPAF-II specific MOIs (Figure 5C). Treatment with ruxolitinib resulted in almost complete killing of all HPAF-II clones at all MOIs. In the absence of ruxolitinib, knockdown of MX1 significantly improved killing compared to the original HPAF-II and scramble-shRNA control clone. As in the Figure 5B experiment, the effect of MX1 KD alone was lower than the effect of ruxolitinib alone (Figure 5C). This is not surprising considering that a number of ISG are downregulated by this JAK1/2 inhibitor, many of which have potential antiviral activity. Therefore, it is likely that a combination of the identified ISGs collectively contributes to resistance.

### Genomic biomarkers of resistance to VSV

Genomic mutations of oncogenes and tumor suppressor genes have been shown to affect viral tropism. For example, mutation to Cyclin-Dependent Kinase Inhibitor 2A (CDKN2A) facilitates herpes simplex virus (HSV) replication in cancer cells [27]. Conversely, knockdown of the retinoblastoma (Rb) tumor suppressor reduced replication efficiency of human cytomegalovirus (HCMV) [28]. Important to our study, mutation of tumor suppressor gene p53 can disrupt the antiviral type I IFN signaling pathway [29, 30]. Other examples with different oncolytic viruses also link oncogenic KRAS mutations, which are initiating mutations in PDAC, to downregulation of ISGs in multiple cancer types [31–33]. To determine if a particular oncogene or tumor suppressor profile is associated with resistance or permissiveness of PDACs to VSV, we isolated genomic DNA from the resistant (HPAF-II and Hs766T) and permissive (Mia PaCa-2 and Capan-1) cells lines, and searched for more than 2,800 mutations in the Catalogue of Somatic Mutations in Cancer (COSMIC) mutations of 50 oncogenes and tumor suppressors using Ion Ampliseq Hotspot analysis (Table 3; complete listing is given in Supplementary Table S5). Importantly, this assay did not allow for entire genome analysis and focused only on 50 common tumor suppressors or oncogenes. Table 3 shows the predicted damaging genomic mutations found in all 4 cell lines. Consistent with the previous studies, all PDAC cell lines had common KRAS mutations, a known initiating mutation of PDAC. Additionally, HPAF-II, MIA PaCa-2, and Capan-1 have TP53 mutations. As shown in our previous study, Hs766T has a TP53 deletion that Ion Ampliseq reports as “wild-type” [20]. Interestingly, different TP53 mutations were detected in the resistant HPAF-II cell line compared to the permissive cell lines MIA PaCa-2 and Capan-1 mutations (Table 3). In addition, the MET Proto-Oncogene, Receptor Tyrosine Kinase (MET) p.S178S mutation was found only in permissive cells, and the Neurogenic Locus Notch Homolog Protein 1 (NOTCH1) p.Q2459K mutation was found in resistant cells versus the p.L2457V found in permissive cells. To examine if these genomic signatures indeed correlated with PDAC phenotype in regard to VSV, we expanded this analysis to a larger set of 7 additional PDAC cell lines as well as 2 non-malignant pancreatic ductal cell lines. Such analysis of a larger set of PDAC cell lines showed that these putative correlations do not hold up (Supplementary Table S5). In general, no clear correlation was found between the assayed genomic mutations and resistance of PDACs to VSV-ΔM51, although the analysis generated valuable genotypic data for future studies. As this study was limited to the most common cancer associated mutations, future genomic analysis may identify changes to other genes (e.g., ISGs) that could be correlated with resistance of PDACs to VSV- ΔM51.

**Table 3:** Damaging genomic mutations in 50 cancer-related genes identified using Ion AmpliSeq Cancer Hotspot Panel

	RESISTANT CELL LINES				PERMISSIVE CELL LINES			
	HS766T		HPAF II		Mia PaCa2		Capan-1	
Gene	Translation Impact	Gene region	Translation Impact	Gene region	Translation Impact	Gene region	Translation Impact	Gene region
ABL1	p.G383G; p.G402G	E						
APC	p.K1444R; p.K1462R	E	p.K1444R; p.K1462R	E	p.K1444R; p.K1462R	E		
APC	p.K1462K; p.K1444K	E	p.K1462K; p.K1444K	E	p.K1462K; p.K1444K	E		
APC	p.T1475T; p.T1493T	E / B	p.T1475T; p.T1493T	E / B	p.T1475T; p.T1493T	E / B	p.T1475T; p.T1493T	E / B
APC			p.K1292K; p.K1310K	E				
CSF1R						U		U
EGFR	p.Q787Q	E / B	p.Q787Q	E / B	p.Q787Q	E / B	p.Q787Q	E / B
ERBB4				I				
FGFR3			p.T539T; p.T653T; p.T651T	E	p.T539T; p.T653T; p.T651T	E	p.T539T; p.T653T; p.T651T	E
FLT3		E		I		I		I
GNAS				I				
HRAS	p.H27H	E / B			p.H27H	E / B	p.H27H	E / B
KDR			p.Q472H	E / B				
KDR				I		I		I
KIT	p.K542K; p.K546K	E / B						
KRAS	p.Q61H	E / P	p.G12D	E / P	p.G12C	E / P	p.G12C	E / P
MET	p.A179T	E			p.S178S	E / B	p.S178S	E / B
NOTCH1	p.Q2459K	E	p.Q2459K	E	p.L2457V	E / LP	p.L2457V	E / LP
PDGFRA	p.P567P	E / B	p.P567P	E / B	p.P567P	E / B	p.P567P	E / B
PIK3CA				I				
RET			p.L769L	E / B	p.L769L	E / B	p.L769L	E / B
RET			p.S904S	E / B	p.S904S	E / B	p.S904S	E / B
SMARCB1				I				
TP53			p.P151S; p.P112S; p.P19S	E / P	p.R209W; p.R116W; p.R248W	E / P	p.R209W; p.R116W; p.R248W	E / P
TP53			p.P72R; p.P33R	E /B				

Conducted on resistant (HPAF-II and Hs766T) and permissive (MIA Paca-2 and Capan-1) cells lines. Translation impact signifies an amino acid difference from wild type while Gene Region denotes where the mutation was detected: E=exonic, I=intronic, U=3′UTR; Pathogenicity: B=benign, P=pathogenic, LP=likely pathogenic.

## DISCUSSION

This is the first study to conduct global transcriptome analysis PDAC cell lines treated with ruxolitinib and TPCA-1, two drugs that dramatically increase permissiveness to VSV-ΔM51 infection and oncolysis in otherwise resistant cell lines [16]. Ruxolitinib (a specific JAK1/JAK2 inhibitor) was highlighted here based on its use in current phase 1, 2, and 3 clinical trials against pancreatic cancer (trials NCT01423604, NCT02117479, NCT02119663, and NCT01822756). We were also interested in comparing effect of ruxolitinib to TPCA-1, which potentially could be used therapeutically as a dual inhibitor of IKK-β and JAK1 [16]. As an inhibitor of both JAK1 and JAK2, key signaling molecules in multiple cellular pathways, ruxolitinib has the potential to impact the expression of thousands of genes. Instead it had a surprisingly modest effect, changing the expression of only 38 genes in both cell lines combined, most of which were ISGs. As expected for a drug targeting additional pathways, TPCA-1 affected expression of a much larger number of genes, but downregulated many of the same ISGs as ruxolitinib, suggesting that these common targets are behind the shared enhancement of viral replication by both ruxolitinib and TPCA-1 [16].

Additionally, our study allowed us to identify a set of putative biomarkers of cellular resistance to VSV, all of which are ISGs. In particular, 8 genes (Mx1, EPSTI1, XAF1, IFI44L, GBP1, SAMD9, SAMD9L, and CMPK2) were expressed in the highly resistant cell lines HPAF-II and Hs766T but not the highly permissive cell lines MIA PaCa-2 and Capan-1 in the absence of virus infection. Six of these genes (MX1, EPSTI1, XAF1, GBP1, SAMD9, and SAMD9L) were confirmed in these 4 cell lines not only on the RNA level, but also on the protein level. When we expanded our analysis to 10 human PDAC cell lines, most of these genes are overexpressed only in highly resistant HPAF-II and Hs766T cell lines. However, we also observed expression of SAMD9 and SAMD9L in VSV-permissive AsPC-1, Capan-2, HPAC, and T3M4 cell lines, indicating that these genes cannot be used individually as biomarkers of PDAC resistance to VSV. Nevertherless, the analysis of the 10 PDAC cell lines demontsrate the constitutive co-expression of all 6 potential biomarkers (MX1, EPSTI1, XAF1, GBP1, SAMD9, and SAMD9L) in VSV-resistant, but not in VSV-permissive PDACs.

While we have shown previously that MX1 is constitutively expressed in resistant cell lines [15], the remaining 7 genes are a novel finding regarding PDAC resistance to OV therapy. All of these genes have been shown previously to have antiviral activity against various viruses: MX1 [34], Epithelial Stromal Interaction Protein 1 (EPSTI1) [35], Sterile Alpha Motif Domain Containing 9 (SAMD9) [36], Sterile Alpha Motif Domain Containing 9-Like (SAMD9L) [37], Guanylate Binding Protein 1 (GBP1) [38] and Cytidine Monophosphate (UMP-CMP) Kinase 2 (CMPK2) [39], Interferon-Induced Protein 44-Like (IFI44L) [40], and XIAP Associated Factor 1 (XAF1) [41]. Importantly, 3 of these genes (MX1, GBP1, and CMPK2) have specifically been shown to play a role in resistance of cells to VSV [15, 38, 39]. While MX1 is known to interfere with VSV transcription initiation [25], the role for the other proteins in anti-VSV activity is not as clear. GBP1 has been shown to promote IFN production in response to VSV infection [38], and CMPK2 has been found in purified VSV preparations and is thought to have activity associated with the VSV large (L) or nucleocapsid (N) proteins [39]. The remaining genes, to the best of our knowledge, have not been specifically linked to VSV infection. However, XAF1 is involved in the apoptotic pathway, which may be important for the VSV life cycle [42]. The role for each of these putative biomarkers in antiviral activity against VSV is beyond the scope of this study, but further investigation into how they influence the virus life cycle would be important in regard to basic VSV biology and clinical applications.

The known antiviral effects of these ISGs along with the fact that ruxolitinib effectively breaks resistance to VSV-ΔM51 while affecting very few non-ISGs suggests that the constitutive expression of these genes is likely a causative rather than merely correlative factor. Furthermore, we have previously shown that WT VSV, which is capable of inhibiting host antiviral responses but cannot be used clinically due to its neurotoxicity, more effectively kills resistant cell lines than VSV-ΔM51 [14], also consistent with causation. To determine if KD of the identified genes would indeed facilitate virus replication, we conducted a proof of principle study using shRNA-mediated KD of MX1. Our results show a partial reversal of resistance. This finding demonstrates that these putative biomarkers may play a synergistic role in resistance and that a combinational KD approach may be required to achieve optimal efficacy. Future studies will examine a combinational KD on virus replication.

Intriguingly, treatment with ruxolitinib or TPCA-1 resulted in downregulation of a distinct subset of the hundreds of known ISGs. It is likely that only some ISGs are constitutively expressed in VSV-ΔM51 resistant PDAC cell lines rather than ruxolitinib and TPCA-1 specifically target certain ISGs for downregulation [15]. Cancer cells selected for radiation damage resistance were shown to constitutively express select ISGs in a manner associated with STAT1 overexpression [43]. This set overlaps with that seen in our present study. Further, upregulation of ISGs like STAT1, STAT2, or IRF9 is known to promote cancer resistance to chemotherapy and radiotherapy as demonstrated in human head and neck squamous cell carcinoma and breast cancer [43–46]. Chronic exposure to type I IFN has also been shown to cause expression of only a subset of ISGs (again with significant overlap with that seen here) and to lead to resistance to DNA damaging agents as well as virus infection [47]. It has been suggested that chronic type I IFN exposure may occur naturally in cancer cells as a result of environmental insults, mutations leading to dysregulation of interferon production, or continual stimulation of the type I IFN pathway by damage associated molecular patterns caused by the cancerous state [47]. Overall this suggests a possible mechanism for constitutive ISG expression and VSV-ΔM51 resistance in PDAC cell lines although this remains to be experimentally determined in future studies. Our work extends the observations made in regard to chemotherapy and radiation resistance to OV therapy and suggests there may be a common mechanism and set of biomarkers. It will be interesting to expand this study to other cancer types and viruses to determine similarities or differences in the ISG profiles of resistance cells.

## MATERIALS AND METHODS

### Virus, cell lines, and inhibitors

The recombinant VSV-ΔM51-GFP (referred to in this study as VSV-ΔM51) has been described previously [5], and was kindly provided by Jack Rose (Yale University). VSV-ΔM51 has a deletion of the methionine at amino acid position 51 of the matrix protein, and the green fluorescent protein (GFP) ORF inserted between the VSV G and L genes. The following human PDAC cell lines were used in this study: AsPC-1 (ATCC CRL-1682), Capan-1 (ATCC HTB-79), Capan-2 (ATCC HTB-80), CFPAC-1 (ATCC CRL-1918), HPAC (ATCC CRL-2119), HPAF-II (ATCC CRL-1997), Hs766T (ATCC HTB-134), MIA PaCa-2 (ATCC CRL-1420), Panc-1 (ATCC CRL-1469), Suit2 [48] and T3M4 [49]. Also, a non-malignant human pancreatic duct epithelial (HPDE) cell line [50] was used, that was previously generated by introduction of the E6 and E7 genes of human papillomavirus 16 into normal adult pancreas epithelium [50]. In addition, the non-malignant human pancreatic Nestin-expressing hTERT-HPNE (ATCC CRL-4023) was a gift from Dr. Anirban Maitra (Johns Hopkins) and maintained in ATCC complete media [51]. After receipt, the human origin of all PDAC cell lines as well as HPDE and hTERT-HPNE cell lines was confirmed by partial sequencing of KRAS and actin. As expected, all PDAC cell lines had a mutation in KRAS, as is typical for PDACs (data not shown). Additionally, the current study demonstrates PDAC-specific mutations in PDAC cell lines but not in HPDE and hTERT-HPNE cells lines (Supplementary Table S5). The baby hamster kidney BHK-21 fibroblast cell line (ATCC CCL-10) was used to grow virus and determine VSV-ΔM51 titers. Capan-1, CFPAC-1, HPAC, MIA PaCa-2, Panc-1, Hs766T, and Suit2 cells were maintained in Dulbecco's modified Eagle's medium (DMEM, Cellgro); AsPC-1, Capan-2, and T3M4 in RPMI 1640 (HyClone); HPAF-II and BHK-21 in modified Eagle's medium (MEM, Cellgro); HPDE in Keratinocyte-SFM (K-SFM, Gibco); hTERT-HPNE in 75% DMEM (without glucose) and 25% Medium M3 Base (Incell Corp.), then supplemented with 5% FBS, 10 ng/ml human recombinant EGF and 5.5 mM D-glucose and 750 ng/ml puromycin. Unless specified above, all cell growth media were supplemented with 9% fetal bovine serum (FBS, Gibco), 3.4 mM L-glutamine, 900 U/ml penicillin and 900 μg/ml streptomycin (HyClone). MEM was additionally supplemented with 0.3% glucose (w/v). K-SFM was never supplemented with serum. Cells were kept in a 5% CO2 atmosphere at 37°C. For all experiments, PDAC cell lines were passaged no more than 10 times. TPCA-1 was purchased from Tocris Bioscience. Ruxolitinib (INCB018424, trade names Jakafi and Jakavi) was purchased from Selleck Chemicals.

### Virus replication and cell viability assays

Cells were seeded in 96-well plate so that they reached approximately 80% confluence after 24 hours (h). Cells were mock infected or infected with VSV-ΔM51 in DMEM without FBS at the specified multiplicity of infection (MOI, based on virus titer on BHK-21 cells). Virus-containing media was aspirated after 1 h absorption period, and replaced with growth media containing 5% FBS and either mock-treated or treated with inhibitor (here and elsewhere mock treatment and inhibitor treatment contained 0.3% DMSO). After infection, virus-driven GFP fluorescence was measured at regular intervals (CytoFluor Series 4000, excitation filter of 485/20 nm, emission 530/25 nm, gain=63; Applied Biosystems). Cell viability was analyzed by a 3-(4,5-dimethyl-2-thiazolyl)-2,5-diphenyl-2H-tetrazolium bromide (MTT) cell viability assay (Sigma-Aldrich).

### RNA microarray analysis

HPAF-II and Hs766T cells (in triplicate) were seeded in 6-well plates so that they reached approximately 80% confluence at 24 h. Cells were washed with PBS, and then mock treated (with serum-free DMSO) or treated with 2.5 μM ruxolitinib or 8 μM TCPA-1 in serum-free DMSO for 6 h. Cellular RNA was extracted with TRIzol (Life Technologies) per the manufacturer protocol with slight modification. In brief, following the first phase separation, the aqueous layer was transferred to a new tube. Then, 500 μl of TRIzol and 100 μl of chloroform were added and phase separation was repeated. Isolated RNA was run on a Bioanalyzer 2100 (Agilent) to check for purity. RNA integrity number (RIN) values were ≥ 7. Samples were reverse transcribed, amplified and labeled using the 3′ IVT Express Kit (Affymetrix). The resultant labeled complementary RNA (cRNA) was purified and fragmented as per vendor's instructions. The cRNA samples together with probe array controls were hybridized onto Affymetrix Human Genome U133+ Plus PM array strips, which cover more than 47,000 transcripts and variants selected from GenBank, dbEST, and RefSeq. Hybridization controls were spiked into the cRNA samples to monitor and troubleshoot the hybridization process. Probes for housekeeping genes were used to assess sample integrity. Hybridization, washing, staining and scanning were performed using Affymetrix GeneChip system instruments. Affymetrix GeneAtlas instrument control software version 1.0.5.267 was used to analyze microarray image data and to compute intensity values. Affymetrix .CEL files containing raw, probe-level signal intensities were analyzed using Partek Genomics Suite version 6.6.12.0713 (Partek). Robust multichip averaging (RMA) was used for background correction, quantile normalization and probe set summarization with median polish (195). Statistical difference was calculated by two-way ANOVA analysis with a false discovery rate (FDR) of 0.05. Based on the Interferome Database v2.01, genes with at least a 10-fold increase in expression in human tissue upon type I interferon stimulation under at least 2 experimental conditions were considered to be Type I Interferon stimulated ISGs. Microarray data were deposited to the ArrayExpress database (accession E-MTAB-4576, “Global effects of ruxolitinib and TPCA-1 on cellular gene expression”).

### RNA RT-PCR analysis

Cells were seeded in a 6-well plate as described above and treated with the specified inhibitor for 24 h before infection. Cells were then mock infected or infected with VSV-ΔM51 in DMEM without FBS at a MOI of 10 (based on VSV-ΔM51 titer on BHK-21 cells). Virus-containing media was aspirated after 1 h absorption period, and replaced with growth media containing 5% FBS and the same treatment as prior to infection. Cells were harvested 12 h post-infection (p.i.) and total RNA was extracted using TRizol as per manufacturer instructions (Ambion), and reverse transcribed using SMART-Scribe reverse transcriptase (Clontech Laboratories, Inc.) as per manufacturer's protocol. PCR products were electrophoresed on a 2% agarose gel with ethidium bromide and photographed using a GelDoc-It imager (UVP Imaging). Primers used for PCR are shown in Supplementary Table S1.

### Western blot analysis

Cells were seeded in 6-well plates so that they reached approximately 80% confluence at 24 h after seeding. Cells were then mock treated or treated with 2.5 μM ruxolitinib for 24 h before infection. Next, cells were mock infected or infected with VSV-ΔM51 in DMEM without FBS at an MOI of 10 (based on virus titer on BHK-21 cells). Virus-containing media was aspirated after 1 h absorption period, and replaced with growth media containing 5% FBS and the same treatment as prior to infection. Cells were harvested at 16 h p.i. To determine VSV replication levels in all PDAC cell lines, cells were mock infected or infected with VSV-ΔM51 in DMEM without FBS at an MOI of 5 (based on virus titer on Suit-2 cells). Cells were harvested at 8 h p.i. Media was removed and cells were lysed using 0.0625 M Tris-HCl (pH 6.8), 10% glycerol, 2% SDS, 5% 2-mercaptoethanol, and 0.02% (w/v) bromophenol blue. Total protein was separated by electrophoresis on SDS-PAGE gels and electroblotted to polyvinylidene difluoride (PVDF) membranes. Membranes were blocked using 5% non-fat powdered milk in TBS-T [0.5 M NaCl, 20 mM Tris (pH 7.5), 0.1% Tween20]. Membranes were incubated in TBS-T with 5% BSA or milk with 0.02% sodium azide and 1:5000 rabbit polyclonal anti-VSV antibodies (raised against VSV virions), 1:1000 rabbit anti-MX1 (Sigma-Aldrich, SAB1100070), 0.2 μg/ml rat monoclonal anti-GBP1 (Sigma-Aldrich, SAB4200056), 1:500 anti-SAMD9L (Sigma-Aldrich, HPA019461), 1 μg/ml mouse monoclonal anti-EPSTI1 (Abnova, H00094240-M02A), 1:1000 rabbit monoclonal anti-XAF1 (Cell Signaling, #13805), 1:1000 rabbit polyclonal anti-SAMD9 (Thermo Fisher, PA5-25613). In addition, the following antibodies were tried without success: 1 μg/ml rabbit polyclonal (PA5-34461) and 1:1000 μg/ml (PA5-25130) against CMPK2, and 1:500 mouse polyclonal (Abnova, H00010964-A01) and 1:200 rabbit polyclonal (Santa Cruz, sc-101981) against IFI44L. The 1:2000 goat anti-mouse or 1:2000 goat anti-rabbit or 1:2000 goat anti-rat horseradish peroxidase-conjugated secondary antibodies (Jackson-ImmunoResearch) were used. The Amersham ECL Western Blotting Detection Kit (GE Healthcare) was used for detection. To verify total protein in each loaded sample, membranes were re-probed with mouse anti-actin antibody (Thermo Fisher, MA5-15739) or rabbit anti-GAPDH antibody (Santa Cruz, sc-25778) or stained with Coomassie Blue R-250.

### Production of stable MX1-shRNA HPAF-II cells

For stable knockdown of MX1 expression, a pLKO.1-puro plasmid-based shRNA targeting the sequence CCTCTATTACTGAATGGAGAT or GCTTTGTGAATTACAGGACAT was employed (MX1-shRNA1 and MX1-shRNA3, respectively). Additionally, a scramble-shRNA plasmid CAACAAGAT GAAGAGCACCAA was used as a control. A primer containing the target sequence along with a stem loop followed by the reverse target sequence was annealed to a complimentary primer and inserted into the EcoRI and AgeI sites of the pLKO.1-puro plasmid, which was a gift from David Root (Addgene #10878) [52]. Lentiviral particles were produced via TRANSIT-TKO (Mirus)-mediated triple transfection of AD293 cells with MX1-shRNA1, MX1-shRNA3, or the scramble-shRNA plasmid along with the lentiviral envelope plasmid pMD2.G and the lentiviral packaging plasmid psPAX2. Both pMD2.G (Addgene #12259) and psPAX2 (Addgene #12260) were a gift from Didier Trono. HPAF-II cells were transduced with MX1-shRNA1, MX1-shRNA3, or scramble-shRNA containing lentiviral particles and stable clones were selected using 6 μg/mL puromycin.

### Genomic mutation profiling

Cellular genomic DNA was isolated from PDAC cell lines using the Life Technologies Purelink Genomic DNA isolation kit. The Ion AmpliSeq™ Cancer Hotspot Panel v2 Kit (Life Technologies) containing 207 primer pairs was used to perform multiplex PCR for the preparation of amplicon libraries from genomic hot spot regions that are frequently mutated in human genes associated with cancer, including approximately 2800 Catalogue of Somatic Mutations in Cancer (COSMIC) mutations of 50 oncogenes and tumor suppressor genes. Sequencing libraries were prepared using an Ion AmpliSeq Library Kit (Life Technologies) per manufacturer's instructions. Briefly, amplicons were ligated to Ion-compatible adapters, followed by nick repair to complete the linkage between adapters and DNA inserts. The libraries were clonally amplified by emulsion PCR on Ion Sphere Particles (ISPs) using the Ion OneTouch 200 Template Kit (Life Technologies) as directed. Following amplification, the template-positive ISPs were enriched to maximize the number of sequencing reads produced using the Ion PGM Sequencing 200 Kit (Life Technologies) on an Ion PGM Sequencer (Life Technologies) and Ion 314 Chips (Life Technologies). Raw data was transferred to the Ion PGM Torrent Server for base calling, preprocessing 3′ trimming, quality control and assessment, and mapping. Variant calling and annotation was performed using Ion Reporter Software (Life Technologies) and Ingenuity Variant Analysis Knowledge Module (Ingenuity Systems) for Ion Reporter.

## SUPPLEMENTARY TABLES












