# Supplementary material for: Novel biomarkers of resistance of pancreatic cancer cells to oncolytic vesicular stomatitis virus
Source: Oncotarget. 2016 Aug 11;7(38):61601–18. doi: 10.18632/oncotarget.11202 (PMC5308675; doi:10.18632/oncotarget.11202)
Supplement: Supplementary file 1 [file oncotarget-07-61601-s001.pdf]

Novel biomarkers of resistance of pancreatic cancer cells to oncolytic vesicular stomatitis virus

SUPPLEMENTARY TABLES

Supplementary Table S1: List of target genes and oligonucleotides used in the study.

See Supplementary File 1

Supplementary Table S2: All genes with at least a 2-fold difference in expression between untreated Hs766T and untreated HPAF-II cells.

See Supplementary File 2

Supplementary Table S3: All genes with at least a 2-fold difference in expression between drug treated and untreated cells.

See Supplementary File 3

Supplementary Table S4: All genes with at least a 1.5-fold difference in expression between drug treated and untreated cells.

See Supplementary File 4

Supplementary Table S5: Total detected genomic mutations in 50 cancer-related genes identified using AmpliSeq Cancer Hotspot Panel.

See Supplementary File 5
